# IMU-Based Analysis of Task-Dependent Associations Between Anticipatory Postural Adjustments and Gait Speed Under Dual-Task Conditions

**DOI:** 10.3390/s26175630

**Published:** 2026-09-04

**Authors:** Yusuke Sakaki, Hiromasa Akagi, Ami Kawata, Daisuke Sawamura, Hiroki Mani, Naoya Hasegawa

**Affiliations:** 1 Graduate School of Health Sciences, Hokkaido University, Kita 12 Nishi 5, Kita-Ku, Sapporo 060-0812, Hokkaido, Japanchiroru259@eis.hokudai.ac.jp (A.K.); 2 Department of Rehabilitation, Sapporo Nishimaruyama Hospital, 7-25, Maruyama Nishimachi 4, Chuo-Ku, Sapporo 064-0944, Hokkaido, Japan; 3 Department of Rehabilitation Sciences, Faculty of Health Sciences, Hokkaido University, Kita 12 Nishi 5, Kita-Ku, Sapporo 060-0812, Hokkaido, Japan; D.sawamura@pop.med.hokudai.ac.jp; 4 Department of Physiotherapy, Faculty of Medicine, Dentistry and Health Sciences, University of Melbourne, Grattan Street, Parkville, VIC 3010, Australia; 5 Faculty of Welfare and Health Science, Oita University, 700, Dannoharu, Oita 870-1192, Oita, Japan; mani-hiroki@oita-u.ac.jp

**Keywords:** anticipatory postural adjustments, dual-task, gait initiation, gait speed, inertial measurement units

## Abstract

**Highlights:**

**What are the main findings?**

- Anticipatory postural adjustments prior to gait initiation are associated with gait speed during dual-task walking.
- Anteroposterior amplitude is associated with gait speed only under dual-task conditions, whereas duration is associated under both single- and dual-task conditions.

**What are the implications of the main findings?**

- Cognitive–motor demands modify how anticipatory control processes are related to gait performance.
- Inertial measurement units enable the detection of task-dependent changes in anticipatory postural adjustments.

**Abstract:**

Anticipatory postural adjustments (APA) generate initial center of mass motion during gait initiation and are associated with gait performance. While APA amplitude and duration have been linked to gait speed under single-task conditions, it remains unclear how these relationships are altered when motor control is constrained by cognitive demands. This study aimed to examine the association between APA characteristics prior to gait initiation and gait speed under dual-task conditions with externally paced cognitive load. Thirty-one healthy young adults performed fast walking under single- and dual-task conditions. Gait speed and APA parameters, and first-step range of motion were assessed using inertial measurement units. To examine associations between APA parameters and gait speed, multiple regression analyses were conducted separately for each condition. Longer APA duration was associated with decreased gait speed under both conditions. Greater anteroposterior amplitude of APA was associated with increased gait speed only under the dual-task condition, whereas no such association was observed under the single-task condition. No significant association was observed between cognitive performance and gait speed. These findings indicate that the association between APA characteristics and gait speed differs depending on task demands, suggesting that cognitive-motor constraints may modify the role of anticipatory control in gait performance.

## 1. Introduction

Gait initiation requires the generation of center of mass (COM) motion to transition from a stable standing posture to steady-state walking. This transition is triggered by a backward and lateral shift in the center of pressure (COP), which moves the COM forward and toward the stance limb [1,2,3]. This COP displacement prior to voluntary movement is defined as anticipatory postural adjustments (APA) [1,3,4,5,6]. The backward COP shift contributes to forward propulsion [4,6], whereas the lateral shift toward the swing limb helps to maintain postural stability [5]. The magnitude and timing of APA influence the dynamics of gait initiation and are thought to be associated with subsequent steady-state gait performance.

Under single-task conditions, both APA amplitude and duration have been associated with gait speed. Individuals with Parkinson’s disease (PD) or stroke often exhibit decreased amplitude of APA, reflected in smaller COP displacements both backward and toward the swing limb prior to gait initiation, which is associated with diminished forward propulsion and increased gait instability [3,7,8,9]. These populations also walk more slowly compared to healthy older adults [10,11]. In addition to the APA amplitude, APA duration, which reflects the time required for weight transfer prior to gait initiation [7,12], is often prolonged in these populations [7,13]. Conversely, healthy young adults demonstrate shorter APA durations when instructed to walk as fast as possible [13]. These findings suggest that both APA characteristics prior to gait initiation are associated with gait speed under single-task conditions.

In daily living, gait is frequently performed under conditions requiring the simultaneous execution of motor and cognitive tasks (dual-task conditions), which impose additional constraints on motor control. Several reviews have reported that dual-task conditions are associated with reduced gait speed, shorter stride length, increased stride time, greater gait variability, and reduced gait stability [14,15]. Among these measures, dual-task gait speed is widely used as an index of locomotor performance and is sensitive to cognitive-motor interference. Divided attention, which refers to the ability to allocate attentional resources across multiple tasks, plays a central role in dual-task gait performance [16]. Increasing cognitive demands in everyday environments, such as those associated with smartphone use, may further challenge the control of gait [17,18]. Therefore, identifying factors associated with gait speed under dual-task conditions is important for understanding locomotor control when motor behavior is constrained by cognitive load. To address this, reliable quantification of movement characteristics during both gait initiation and steady-state gait are required. Wearable inertial measurement units (IMUs) offer a practical approach for capturing such task-dependent changes under real-world conditions.

Despite this, the relationship between APA and gait speed during cognitive-motor interference remains unclear. APA is a prerequisite for appropriate voluntary movement and involves activation of cortical networks associated with motor planning and motor control [19,20]. Dual-task gait has been shown to increase cortical activity, particularly in the prefrontal and supplementary motor areas, compared to single-task gait [21,22,23]. These findings suggest that cognitive load may alter the coordination between anticipatory and ongoing motor control processes, potentially modifying the relationship between APA prior to gait initiation and gait performance. Importantly, controlling the level of cognitive load is critical when examining this relationship. However, previous studies assessing dual-task gait speed have often used cognitive tasks performed at participants’ own pace [10,24,25], which may introduce variability in task prioritization across individuals. This variability can obscure the relationship between APA and gait speed under dual-task conditions. Therefore, using an externally paced cognitive task may reduce this variability and allow a more controlled evaluation of this association.

Therefore, the aim of this study was to examine the association between APA prior to gait initiation and gait speed under dual-task conditions with externally controlled cognitive load using IMUs. Based on previous studies [3,7,9,12,19,20,21,22,23], we hypothesized that APA characteristics would be associated with gait speed not only under the single-task condition but also under the dual-task condition, with differences in their relative contributions depending on task demands.

## 2. Materials and Methods

### 2.1. Participants

Thirty-one healthy young adults (17 males and 14 females, age: 22.7 ± 1.4 years; height 165.6 ± 8.6 cm; weight 56.2 ± 9.0 kg; body mass index 20.8 ± 1.8 kg/m^2^) participated in this study. The inclusion criteria were an absence of any neurological or musculoskeletal disorders. All participants signed informed consent forms approved by the Institutional Review Board of the Faculty of Health Sciences of Hokkaido University (No. 21–23). All works were conducted in accordance with the Declaration of Helsinki (1964).

### 2.2. Equipment

**Figure 1:**
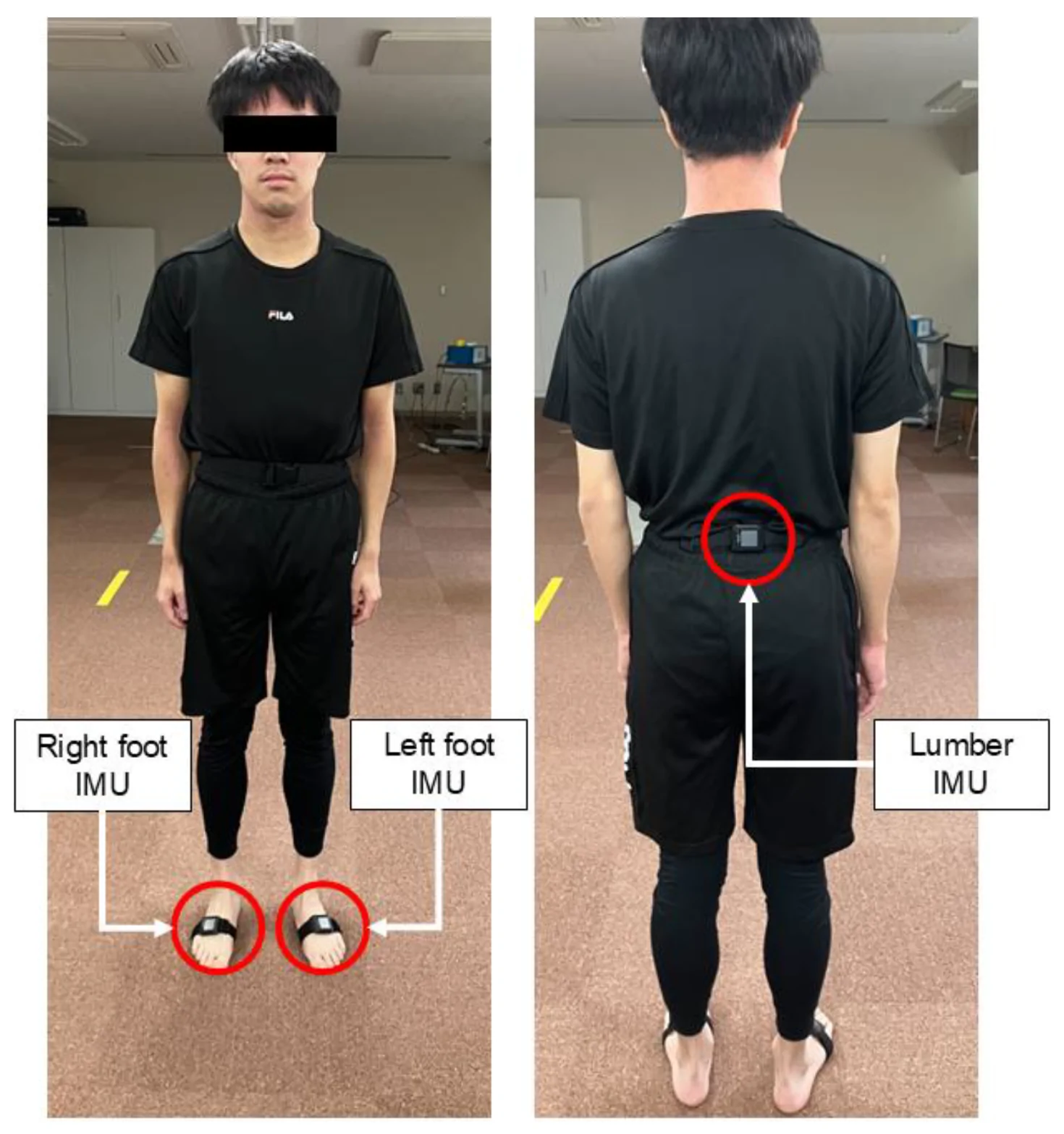
Illustration of the IMU placement. Red circles indicate the locations where the IMUs were attached. All IMUs were secured using Velcro straps.

Previous studies have demonstrated that APA parameters measured using IMUs show validity and reliability comparable to those measured through COP analysis [26,27,28]. Therefore, three Opal sensors (APDM, Portland, OR, USA) were attached to the dorsum of both feet and posterior trunk at the level of L5. The foot sensors were attached with the connector pointing toward the middle toe, with the x-, y-, and z-axes oriented in the anterior–posterior, medio-lateral, and vertical directions, respectively. The lumbar sensor was attached with the connector pointing toward the floor, with the x-, y-, z-axes oriented in the medio-lateral, vertical, and anterior–posterior directions, respectively. All IMUs were positioned according to the manufacturer’s recommendations and secured using Velcro straps to minimize motion between the IMUs and the body segments (Figure 1). The inertial data were collected at a sampling frequency of 128 Hz and streamed to a laptop for automated generation of gait and balance variables using Mobility Lab software (version V2, APDM, Portland, OR, USA). The cognitive task was presented through two speakers (Companion 2; BOSE Co., Framingham, MA, USA) located approximately 3 m anterolaterally to the starting position.

### 2.3. Procedures

Participants were instructed to stand quietly for 30 s and then walk repeatedly back and forth along a straight 7 m walkway as quickly as possible for 60 s. This task was performed under two conditions: a single-task condition, in which participants focused solely on walking, and a dual-task condition, in which participants performed a cognitive task while walking. The order of conditions was fixed: all participants completed the single-task condition first. In this condition, participants were instructed to “walk as quickly as possible” and to “initiate gait as quickly as possible in response to a verbal instruction.” After completing the single-task condition, participants rested in a seated position before beginning the dual-task condition. Based on our previous findings indicating that auditory cognitive tasks exert a greater effect on postural control than visual cognitive tasks, a paced auditory serial addition task (PASAT) was used as the cognitive task in the dual-task condition [29]. In the PASAT, single-digit numbers were presented every 2 s through the speakers, and participants were required to add each new number to the one immediately preceding it [30]. Participants were provided with an explanation of the PASAT and completed a 20 s practice session while seated before performing the dual-task condition. In this condition, participants were instructed to “walk as quickly as possible,” to “initiate gait as quickly as possible in response to a verbal instruction,” and to “respond as quickly and accurately as possible to the cognitive task throughout the dual-task condition, including during quiet standing and walking”. Participants were also asked to allocate attention to both tasks and perform each task as well as possible.

### 2.4. Data Analyses

**Figure 2:**
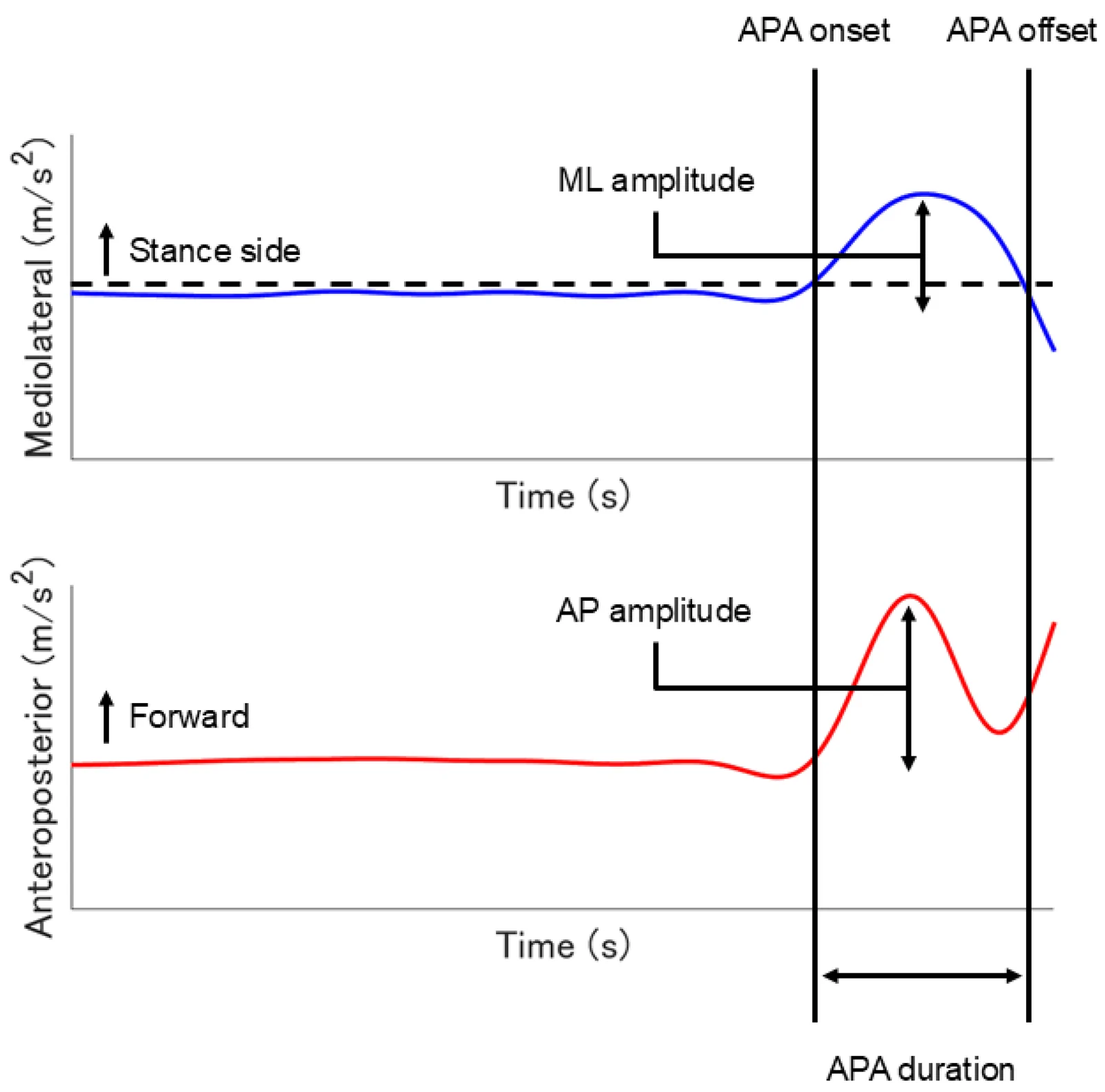
Identification of anticipatory postural adjustments (APA) based on trunk acceleration. The blue time series represents trunk acceleration in the mediolateral (ML) direction, and the red time series represents trunk acceleration in the anteroposterior (AP) direction. The dashed line indicates the threshold for APA onset, defined as the time point at which ML trunk acceleration deviates from baseline by more than three standard deviations. The black vertical lines indicate APA onset and offset. APA parameters extracted from the acceleration profiles—AP amplitude, ML amplitude, and APA duration—are illustrated.

All signals were partially processed automatically using Mobility Lab software and further analyzed offline using MATLAB software (R2019b; MathWorks, Natick, MA, USA). Firstly, APA onset and APA offset were identified (Figure 2). APA onset was defined as the point at which lumber acceleration in the mediolateral (ML) direction deviated from baseline by more than three standard deviations [27,31], with the baseline calculated from the first 3 s of quiet standing. APA offset was defined as the point at which ML lumbar acceleration returned to baseline. The APA phase was defined as the time interval between APA onset and APA offset, which was used as the APA duration. The following APA parameters were then extracted using the Mobility Lab software: (1) anteroposterior (AP) amplitude: peak lumbar acceleration in the AP direction during the APA phase, (2) ML amplitude: the peak lumbar acceleration in the ML direction during the APA phase, and (3) First-step ROM: integrated sagittal angular velocity of the stepping foot from APA offset to initial contact of the step. Signal preprocessing and filtering were performed automatically within the Mobility Lab software. Gait speed was calculated automatically excluding the first step of gait initiation and any steps taken during turning phases.

In the dual-task condition, cognitive performance was assessed by calculating the correct response rate of the PASAT performed during walking.

### 2.5. Statistical Analyses

A paired *t*-test was conducted to compare gait speed between the single- and dual-task conditions, and effect sizes were calculated using Cohen’s *d*. Multiple regression analyses (forced-entry method) were conducted separately for the single- and dual-task conditions to examine the associations between APA parameters and gait speed. Gait speed was used as the dependent variable. Independent variables included AP amplitude, ML amplitude, APA duration, and First-step ROM. For the dual-task condition, PASAT correct response rate was additionally included to account for cognitive performance. Multicollinearity among independent variables was assessed using variance inflation factors (VIFs). In addition, Cohen’s *f*^2^ was calculated to quantify the overall effect size of the regression model. All statistical analyses were performed using SPSS statistics (version 30.0; IBM Co., Armonk, NY, USA). Statistical significance was set at *p* < 0.05.

## 3. Results

Gait speed was significantly lower in the dual-task condition than in the single-task condition (dual-task: 1.59 ± 0.20 m/s; single-task: 1.63 ± 0.17 m/s; *p* = 0.030; *d* = 0.455). The mean correct response rate was 78.0 ± 12.7% in the dual-task condition.

**Figure 3:**
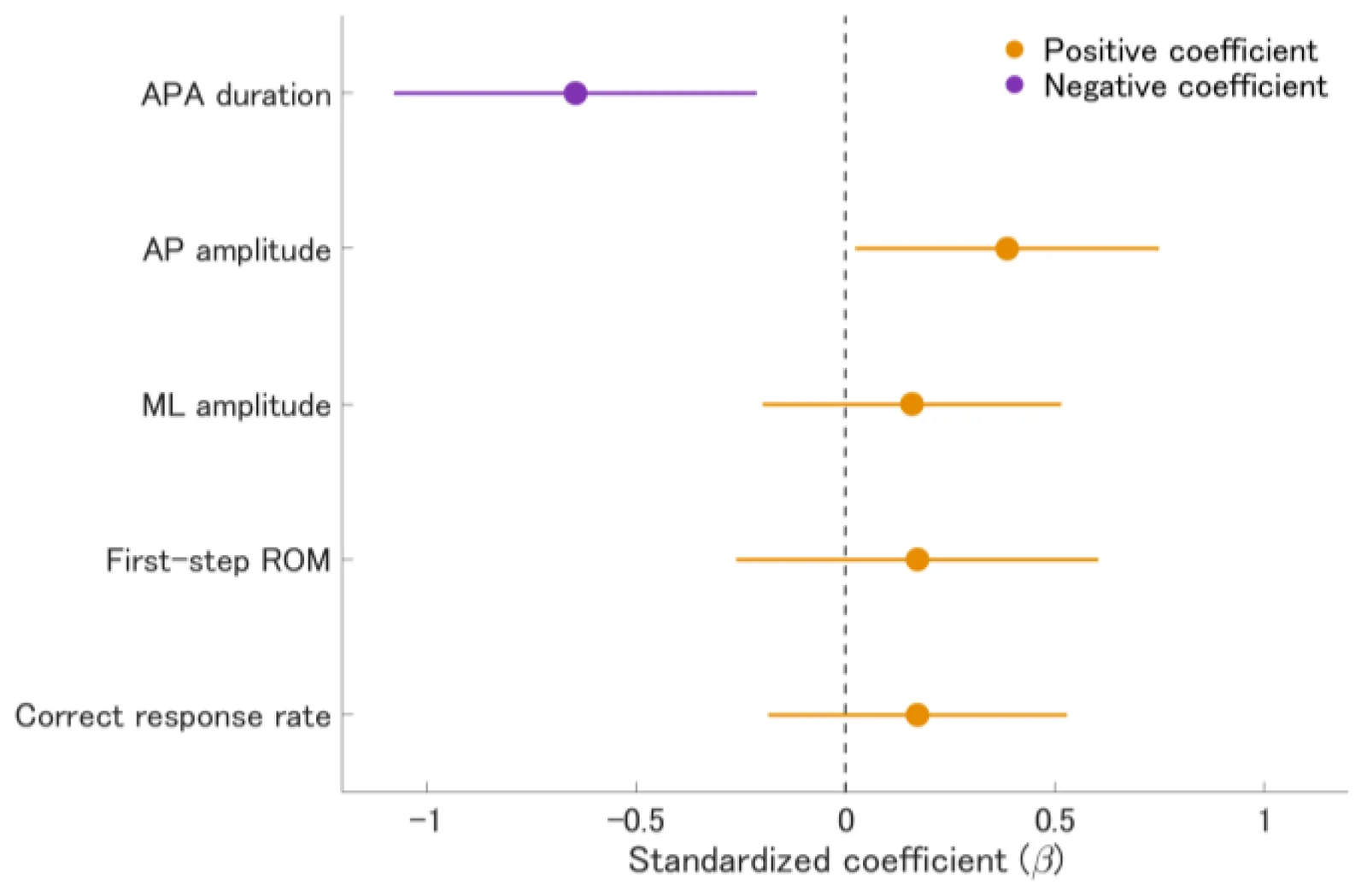
Forest plot of a multiple linear regression analysis predicting gait speed under the dual-task condition. Orange or purple circles represent standardized regression coefficient (*β*). Horizontal bars indicate the 95% corresponding confidence intervals (CIs). The color of the circles reflects the sign of *β*, with orange indicating positive coefficients and purple indicating negative coefficients.

Table 1 summarizes the results of the multiple regression analyses in the dual-task condition. In the dual-task condition, the overall model was significant (*p* = 0.024), explaining 39% of the variance in gait speed (*R*^2^ = 0.387, Adjusted *R*^2^ = 0.265). The model demonstrated a large effect size, as indicated by Cohen’s *f*^2^ of 0.361. Among the predictors, AP amplitude showed a significant positive effect on Gait speed, indicating that larger AP amplitude was associated with increased gait speed (*β* = 0.386, *p* = 0.037; Figure 3). On the other hand, APA duration demonstrated a significant negative effect on Gait speed, meaning that prolonged APA duration was associated with decreased gait speed (*β* = −0.644, *p* = 0.005). ML amplitude, First-step ROM, and the correct response rate of the PASAT were not significant predictors (ML Amplitude: *β* = 0.159, *p* = 0.368; First-step ROM: *β* = 0.172, *p* = 0.421; correct response rate: *β* = 0.172, *p* = 0.331).

**Figure 4:**
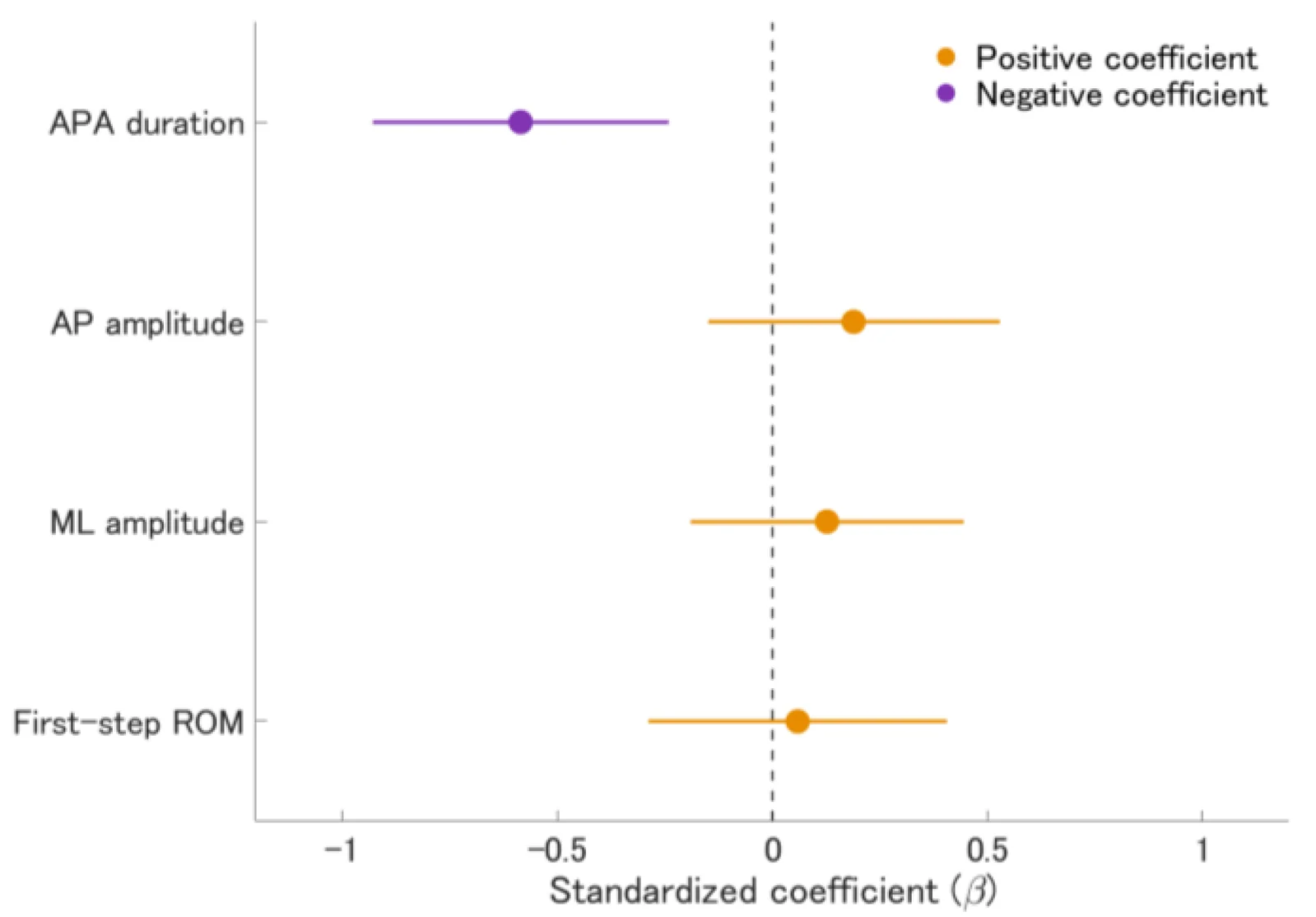
Forest plot of a multiple linear regression analysis predicting gait speed under the single-task condition. Orange or purple circles represent standardized regression coefficient (*β*). Horizontal bars indicate the 95% corresponding confidence intervals (CIs). The color of the circles reflects the sign of *β*, with orange indicating positive coefficients and purple indicating negative coefficients.

In the single-task condition, the overall model was significant (*p* = 0.001), explaining 43% of the variance in the dependent variable (*R*^2^ = 0.430, Adjusted *R*^2^ = 0.342). The model demonstrated a large effect size, as indicated by Cohen’s *f*^2^ of 0.520. Among the predictors, only APA duration demonstrated a significant negative effect on Gait speed (*β* = −0.585, *p* = 0.002; Table 2; Figure 4). Other predictors were not significant (AP amplitude: *β* = 0.189, *p* = 0.260; ML amplitude: *β* = 0.127, *p* = 0.419; and First-step ROM: *β* = 0.059, *p* = 0.730).

## 4. Discussion

This study investigated the association between APA prior to gait initiation and gait speed under dual-task conditions in which cognitive load was externally controlled. Our results showed that APA duration was associated with gait speed under both single- and dual-task conditions, whereas AP amplitude was associated with gait speed only under the dual-task condition. No multicollinearity was observed among the explanatory variables [32], indicating that these associations were statistically stable within the present dataset. These findings suggest that the association between APA characteristics and gait speed differs depending on task demands. Furthermore, no significant association was observed between cognitive performance and gait speed in the dual-task condition, suggesting that variability in cognitive task performance was unlikely to substantially confound the observed relationships. The mean correct response rate of PASAT was 78.0 ± 12.7%, indicating a reasonable degree of variability in task performance. This value is comparable to that previously reported for healthy young adults (80.7 ± 13.3%) [33]. Therefore, the observed accuracy does not appear to indicate either a floor or ceiling effect. Consequently, the absence of a significant association between cognitive performance and gait speed is unlikely to be explained by restricted variability in PASAT performance.

Under both single- and dual-task conditions, prolonged APA duration was associated with decreased gait speed. A previous study has reported that prolonged APA duration reflects less efficient timing of COP and COM displacement during gait initiation [13]. Individuals with PD have been shown to demonstrate prolonged APA duration as well as decreased first-step velocity [8], indicating that motor preparation may be linked to subsequent gait performance. Because first-step velocity increases when individuals walk at higher speeds [34], early-phase locomotor dynamics are associated with steady-state gait speed. Therefore, these findings suggest that APA duration may reflect preparatory processes that are associated with subsequent gait performance, regardless of cognitive load.

Under the dual-task condition, greater AP amplitude was associated with faster gait speed, whereas no such association was observed under the single-task condition. Previous studies reported that AP amplitude, which is defined as the backward COP displacement or peak forward lumbar acceleration, has been linked to the generation of forward movement of the COM required for gait initiation [1,2,3,26,27]. Our previous findings suggest that performing the PASAT may induce a stiffness-like strategy characterized by increased muscle activation during quiet standing [29]. Increased muscle activity has been associated with reduced limits of stability (LOS) [35], reflecting a reduced controllable range of COM displacement [36]. Thus, forward COM motion at gait initiation may be more constrained under the present dual-task condition. Accordingly, greater AP amplitude may be associated with greater forward COM progression under dual-task conditions. Importantly, APA should not be interpreted as directly determining steady-state gait speed, but rather as reflecting initial motor strategies that are associated with subsequent gait performance under specific task constraints. In the present context, cognitive load imposed prior to gait initiation may alter the association between these preparatory strategies and subsequent locomotor behavior. This interpretation is consistent with the view that cognitive-motor interference can alter the coordination between anticipatory and ongoing control processes [16].

In contrast, under the single-task condition, gait speed in healthy young adults may be regulated through multiple mechanisms during walking, including step-by-step adjustments and feedback-based control, thereby reducing reliance on APA amplitude alone. Previous studies have reported associations between AP amplitude and gait speed under single-task conditions in individuals with PD or stroke [3,7,8,9,13,37]. These populations commonly exhibit reduced forward LOS and increased muscle activity during quiet stance [38,39,40,41], which may constrain COM displacement and increase reliance on APA-related mechanisms. Such constraints were not present in the healthy young adults in present study, which may account for the absence of association between AP amplitude and gait speed under the single-task condition.

Although AP amplitude was associated with gait speed under the dual-task condition, ML amplitude was not associated with gait speed in either single- or dual-task condition. ML amplitude is primarily involved in lateral gait stability [5], and reduced ML amplitude has been linked to gait instability in clinical populations [42,43]. In the present study, participants did not exhibit gait instability under either the single- or dual-task condition, which may explain the lack of association between ML amplitude and gait speed. These findings suggest a directional specificity of APA, whereby AP-related components are more closely associated with forward progression, whereas ML components primarily contribute to stability. Furthermore, AP-oriented motor preparation may be more sensitive to cognitive demands than ML stabilization processes [44].

This study has several limitations. First, the sample consisted of a small cohort of healthy young adults, who typically do not exhibit gait instability. Therefore, the influence of instability-related mechanisms may have been underestimated. Consequently, the generalizability of these findings to older adults or individuals with neurological disorders remains unclear, as these populations may exhibit different APA strategies and responses to dual-task demands. Future studies should include older adults or individuals with neurological disorders to clarify how gait instability interacts with APA under externally paced dual-task conditions, and whether the associations observed in the present study differ according to participant characteristics. Second, the order of task conditions was not randomized, as the single-task condition was always performed before the dual-task condition to minimize potential carry-over effects of cognitive strategies associated with the PASAT. Consequently, potential order effects such as fatigue, habituation, or changes in arousal cannot be fully excluded, and the present findings should be interpreted as associations observed under a specific task sequence. Randomized or counterbalanced designs are also needed to confirm the present findings. Third, the regression analyses should be considered exploratory given the sample size relative to the number of predictors. Although multicollinearity was not observed and the observed effect sizes were large, the stability and generalizability of the estimated coefficients may be limited and require confirmation in larger samples. Therefore, the present findings should be interpreted as evidence of associations rather than definitive causal relationships. Finally, electromyography data were not collected. The APA prior to gait initiation is characterized by inhibition of the tonically activities of soleus followed by activation of the tibialis anterior [1,9], and individuals with PD or stroke demonstrate increased activities of tibialis anterior during quiet stance [38,41]. Furthermore, incorporating direct measurements of muscular activity during both quiet standing and gait initiation may provide further elucidate the mechanisms underlying APA under cognitive load.

## 5. Conclusions

In healthy young adults, APA duration and AP amplitude are associated with gait speed under dual-task conditions involving an externally paced cognitive task. These findings suggest that the association between APA characteristics and gait performance may differ under cognitive-motor demands. Furthermore, the present results highlight the potential importance of anticipatory control processes in contributing to gait performance under dual-task conditions. Future studies should examine whether modifying APA characteristics is associated with changes in gait performance under dual-task conditions. In addition, the present findings support the potential utility of IMU-based assessment of APA under dual-task conditions. Accordingly, future studies should investigate these associations under more ecologically valid walking conditions to further establish the clinical applicability and external validity of IMU-based assessment of APA.

## Figures and Tables

**Table 1:** Summary of multiple linear regression analysis predicting gait speed under the dual-task condition.

	Unstandardized		Standardized				
Predictor	B	SE	*β*	CI (Lower/Upper)	*t*-Value	*p*-Value	VIF
**APA duration (s)**	**−1.420**	**0.463**	**−0.644**	**−1.077/−0.212**	**−3.068**	**0.005**	**1.798**
**AP amplitude (m/s^2^)**	**1.735**	**0.789**	**0.386**	**0.024/0.748**	**2.199**	**0.037**	**1.258**
ML amplitude (m/s^2^)	1.482	1.615	0.159	−0.198/0.515	0.918	0.368	1.223
First-step ROM (degree)	0.004	0.005	0.172	−0.260/0.604	0.819	0.421	1.797
Correct response rate (%)	0.265	0.268	0.172	−0.185/0.529	0.991	0.331	1.228

Values in bold indicate significant effect at *p* < 0.05. B, unstandardized coefficient; SE, standard error; *β*, standardized coefficient; CI, 95% confidence intervals; VIF, variance inflation factor.

**Table 2:** Summary of multiple linear regression analysis predicting gait speed under the single-task condition.

	Unstandardized		Standardized				
Predictor	B	SE	*β*	CI (Lower/Upper)	*t*-Value	*p*-Value	VIF
**APA duration (s)**	**−0.831**	**0.238**	**−0.585**	**−0.929/−0.241**	**−3.496**	**0.002**	**1.277**
AP amplitude (m/s^2^)	0.693	0.602	0.189	−0.149/0.528	1.151	0.260	1.235
ML amplitude (m/s^2^)	1.179	1.437	0.127	−0.191/0.445	0.821	0.419	1.090
First-step ROM (degree)	0.001	0.003	0.059	−0.288/0.406	0.349	0.730	1.301

Values in bold indicate significant effect at *p* < 0.05. B, unstandardized coefficient; SE, standard error; *β*, standardized coefficient; CI, 95% confidence intervals; and VIF, variance inflation factor.

## Data Availability

The datasets generated and/or analyzed during the current study are available from the corresponding author on reasonable request.

## Abbreviations

The following abbreviations are used in this manuscript:

APA	Anticipatory Postural Adjustment
AP	AnteroPosterior
COM	Center Of Mass
COP	Center Of Pressure
IMU	Inertial Measurement Unit
MLL	MedioLateral
PASAT	Paced Auditory Serial Addition Task
PD	Parkinson’s Disease
VIF	Variance Inflation Factor
